# An Insight into Microbial Inoculants for Bioconversion of Waste Biomass into Sustainable “Bio-Organic” Fertilizers: A Bibliometric Analysis and Systematic Literature Review

**DOI:** 10.3390/ijms232113049

**Published:** 2022-10-27

**Authors:** Jennifer Michellin Kiruba N, Agnieszka Saeid

**Affiliations:** Department of Engineering and Technology of Chemical Processes, Faculty of Chemistry, Wroclaw University Science and Technology, 50-373 Wroclaw, Poland

**Keywords:** microbial inoculants, bio-organic fertilizers, waste valorization, bioconversion

## Abstract

The plant-microbe holobiont has garnered considerable attention in recent years, highlighting its importance as an ecological unit. Similarly, manipulation of the microbial entities involved in the rhizospheric microbiome for sustainable agriculture has also been in the limelight, generating several commercial bioformulations to enhance crop yield and pest resistance. These bioformulations were termed biofertilizers, with the consistent existence and evolution of different types. However, an emerging area of interest has recently focused on the application of these microorganisms for waste valorization and the production of “bio-organic” fertilizers as a result. In this study, we performed a bibliometric analysis and systematic review of the literature retrieved from Scopus and Web of Science to determine the type of microbial inoculants used for the bioconversion of waste into “bio-organic” fertilizers. The *Bacillus*, *Acidothiobacillus* species, cyanobacterial biomass species, *Aspergillus* sp. and *Trichoderma* sp. were identified to be consistently used for the recovery of nutrients and bioconversion of wastes used for the promotion of plant growth. Cyanobacterial strains were used predominantly for wastewater treatment, while *Bacillus, Acidothiobacillus,* and *Aspergillus* were used on a wide variety of wastes such as sawdust, agricultural waste, poultry bone meal, crustacean shell waste, food waste, and wastewater treatment plant (WWTP) sewage sludge ash. Several bioconversion strategies were observed such as submerged fermentation, solid-state fermentation, aerobic composting, granulation with microbiological activation, and biodegradation. Diverse groups of microorganisms (bacteria and fungi) with different enzymatic functionalities such as chitinolysis, lignocellulolytic, and proteolysis, in addition to their plant growth promoting properties being explored as a consortium for application as an inoculum waste bioconversion to fertilizers. Combining the efficiency of such functional and compatible microbial species for efficient bioconversion as well as higher plant growth and crop yield is an enticing opportunity for “bio-organic” fertilizer research.

## 1. Introduction

Numerous studies have reported that the overuse of conventionally used synthetic fertilizers contributes to the degradation of soil quality in most arable land and negatively impacts its biological community. Although the application of certain chemical fertilizers has improved agricultural yield in response to the increasing demand over the previous decades, now the question of sustainability arises, in the long run, amid decreasing resources and raw materials [1]. This brings about the need to deviate from the traditional linear model of biofertilizer production and adopt circular production from different types of waste-based resources. While the application of synthetic fertilizers since the 1950s has gone a long way in providing food security for many developing countries, they also have downsides due to their extensive overuse.

The inefficient and inappropriate use of chemical fertilizers has led to problems on multiple fronts such as soil quality degradation, groundwater contamination, loss of biodiversity, decrease in soil fertility, resistance development in pests, and acidification issues, among other potential agricultural-based non-point source pollution (NSP) [2,3,4]. On the other hand, several studies propose the replacement of chemical fertilizers with organic, bio-organic, and microbial fertilizers [5,6].

### 1.1. The Need for Bio- and Bio-Organic Fertilizers

Biofertilizers are generally defined as an organism-based composition of micronutrients and carbon substrates such as bacteria, cyanobacteria, fungi, and algae, that can nourish and enhance soil quality [7]. They also have the advantages of being cheap alternatives to synthetic fertilizers, improving crop quality, soil fertility, and food safety while all the while being sustainable. Most common and popular biofertilizers are green manures such as cyanobacterial amendments, bio-formulations of bacteria such as *Azotobacter* sp., *Azospirillum* sp., *Trichoderma* sp., and arbuscular mycorrhizal fungi (AMF) [8]. Apart from microbial “biofertilizers”, organic-based fertilizers are also commonly used by the farming community which includes composting residues such as vermicomposting, agricultural crop residues, and farmyard manure.

The current strategies used to produce biofertilizer bioformulation are the usage of carrier materials such as slurries, granules, powder, and liquid formulations for the dispersal of microbial inoculums targeting the appropriate part of the crop such as root, shoot, or flowers. Microbial inoculums in the form of lyophilisates (powders) or liquid broths (grown in media) are added to various carriers such as clay, peat, and sand, producing a granular or freeze-dried product for distribution [9,10]. The technology of inoculum development for microbial bio-formulations and their carrier substrates are discussed in detail in [11].

### 1.2. The Integration of Circular Economy (CE) in Bio-Organic Fertilizer Production

The burgeoning population with their ever-increasing demand for food and nutritional security substantiates the necessity of biofertilizer research. While in the early stages of biofertilizer research, the raw materials used for bio-formulations such as media supplementation for microbial and algal growth were produced as part of a linear economic model, currently, the production is required to be more sustainable. Various resources such as agro-industrial and municipal wastes are suitable secondary raw materials with appropriate physicochemical composition to be utilized for fertilizer production. According to the CE concept, agro-food industrial wastes and municipal solid waste residues have been extensively studied from the perspective of biorefineries to produce fuels, biochemicals, energy, and biomass. The valorization of waste biomass after pyrolysis or combustion to generate viable and sustainable biofertilizers is being actively pursued [7]. For sustainable use of natural resources used in the production of biofertilizers or organic-based fertilizers as well as to replace the use of chemical fertilizers, biomass generated from food, municipal, and agricultural wastes are processed using composting, aerobic and anaerobic digestion, combustion, pyrolysis and chemical hydrolysis among other technologies [12,13].

#### 1.2.1. Waste Valorization

As discussed above, the enforcement of EU and UN SDG-based policies on circular economy (CE) have led to the development of waste-based biorefineries. This biorefinery concept works on the fundamental principle of waste valorization technologies. Valorization of the wastes considers various types of sources such as food waste, e-waste, agro-industrial wastes, municipal solid waste (MSW), construction and demolition (C&D) debris, medical wastes, textile, plastics and mining associated wastes, which in turn depends on the type of technology applied to convert the waste into a sustainable product such as biomass, biofuel, bioenergy and others [14,15]. A detailed discussion of three major technologies adopted in waste valorization pathways is provided in [16]. One of the pathways includes the search for technology that generates a fuel that not only replaces fossil fuels in terms of supply and demand but also produces less greenhouse gas emissions and carbon footprint. Using waste biomass to produce cleaner energy is one major path favored by environmental-friendly policies. Similarly, the other pathways of waste valorization focus on the biorefinery approach of producing bioplastics, biochemicals, solvents, and biomass from a circular supply chain of waste material using various valorization technologies such as microwave-assisted, pyrolysis, chemical extraction, separation, and isolation techniques, pyrolysis, and bioengineering approaches such as enzymatic, chemo-enzymatic, solid-state fermentation, bioreactor technology, and bioconversion strategy.

#### 1.2.2. Bioconversion

Bioconversion is a biotechnological strategy for waste valorization that exploits the metabolic and enzymatic versatility of a microorganism to produce a sustainable bio-compound such as solvents (acetone), acids (lactic acid, citric acid), bioplastics, biofuels (biodiesel), and other value-added substances post the treatment of waste biomass from any industry through the process of fermentation, digestion or solubilization [17].

This strategy is being extensively applied to convert waste biomass into appropriately formulated biofertilizers. For instance, composting and anaerobic digestion (AD) processes are in effect bioconversion approaches that utilize the metabolic capacity of the thermophilic and decomposer microbial community present in the green manures and lignocellulosic material used in them. Often native and indigenous microbial communities present in a selective habitat possess a unique and specific assortment of enzymatic tools that co-metabolize different substrates (including recalcitrant pollutants) and biotransform metal species to bioavailable forms. However, these localized microbial communities are rather slow and non-optimal, and often require external stimulus through bioaugmentation or biostimulation. Likewise, several studies have tested the bioconversion efficiency of composting and AD processes with the addition of a microbial seed inoculum [18]. Presently, many studies have been conducted to assess the efficiency of microbial fermentation and bio-solubilization to convert other waste materials to fertilizers that specifically target NPK enrichment in soil or plants or produce plant biostimulant components such as humic substances, phytohormones, betaines and bioavailability of micronutrients [19].

#### 1.2.3. Microbial Inoculants

Although microbial inoculants and inoculum technology have a long history in fertilizer and agricultural research, their roles in waste valorization technology for the bioconversion of waste biomass into high-value-added plant biostimulant products are of new and high interest. Bioconversion of secondary raw materials, such as agroindustrial wastes, that are rich in NPK and organic matter necessary for plant growth by the employment of target-specific microorganisms is an evolving field of research. Such a synergistic combination of waste biomass and plant-growth-stimulating microorganisms creates bioorganic fertilizers that reduce dependence on primary raw materials and synthetic fertilizers. They may alleviate issues such as the dispersal of antibiotic resistance and the persistence of recalcitrant residues in current compost and AD-based fertilizers, reducing soil pollution and enhancing crop yield, and soil health [20]. In recent years, the addition or supplementation of microbial inoculants to AD or composting processes have been tested to increase the efficiency of the biorefinery process. Herein, we would like to enlist the research undertaken in the field of bioconversion employed to convert waste biomass into “bio-organic” fertilizer with the aid of substrate-specific microbial inoculant.

Therefore, this study attempts to conduct a bibliometric analysis of biofertilizer research trends during 2012–2021 (the last five years) and a systematic literature review of relevant articles during the same period to determine the microbial inoculants of interest that have been employed for bioconversion of wastes to bio-organic fertilizers.

## 2. Methods

### 2.1. Objectives

The study attempts to track the research trends in biofertilizers in the last five (2017–2021) years, through a bibliometric analysis and systematic literature review of research articles retrieved through specific search strategies from the SCOPUS and Web of Science database.

### 2.2. Search Analysis in Scopus

Searches were conducted in the Scopus and Web of Science database for bibliometric analysis and systematic literature review. The search results were retrieved from the Scopus database using a combination of keywords such as “plant growth”, “fertilizer”, “bioconversion”, “biofertilizers”, “bioinoculant”, “fungi”, “bacteria”, and “waste”. The detailed search strategy is provided in Table 1. The data of the number of articles published by different categories such as journals, year, and country were exported and analyzed.

### 2.3. Bibliometric Analysis by VOSviewer

The bibliographic information of the search results was exported in csv format and analyzed using VOSviewer 4.0 by keywords, co-occurrence patterns of terms, and citation [21]. The bibliographic data was preprocessed during VOSviewer analysis by removing keywords used in the search strategy such as “plant growth”, “fertilizer”, “bioconversion”, “biofertilizers”, “bioinoculant”, “fungi”, “bacteria”, and “waste” to avoid bias in the results. Moreover, during the analysis of index keywords used by the journals, terms such as “priority”, “nonhuman”, and “article” were also cleaned up. Once biased keywords used in the search and retrieval were removed, the weights and scores were set to total link strength and averaged normalized citations, respectively.

### 2.4. Systematic Literature Review

#### 2.4.1. Scope and Definitions

The research question of the study was framed based on PICO [22] and guided by the PRISMA methodology [23]. This review was devised to address the trends in biofertilizer research undertaken during the last five years. The PICO framework is tabulated in Table 2.

#### 2.4.2. Data Retrieval for SLR

**Figure 1:**
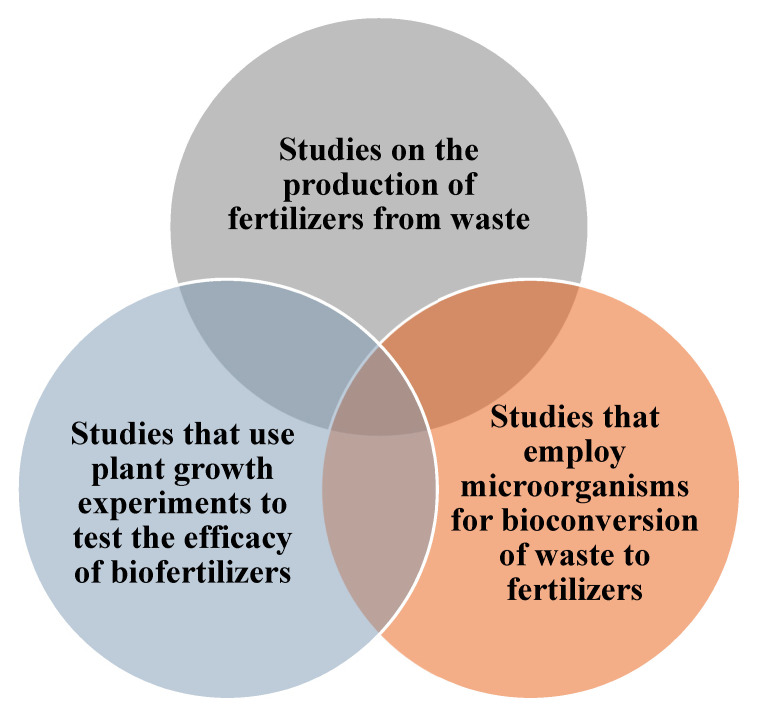
Schematic representation of the inclusion and exclusion criteria utilized in this study.

**Figure 2:**
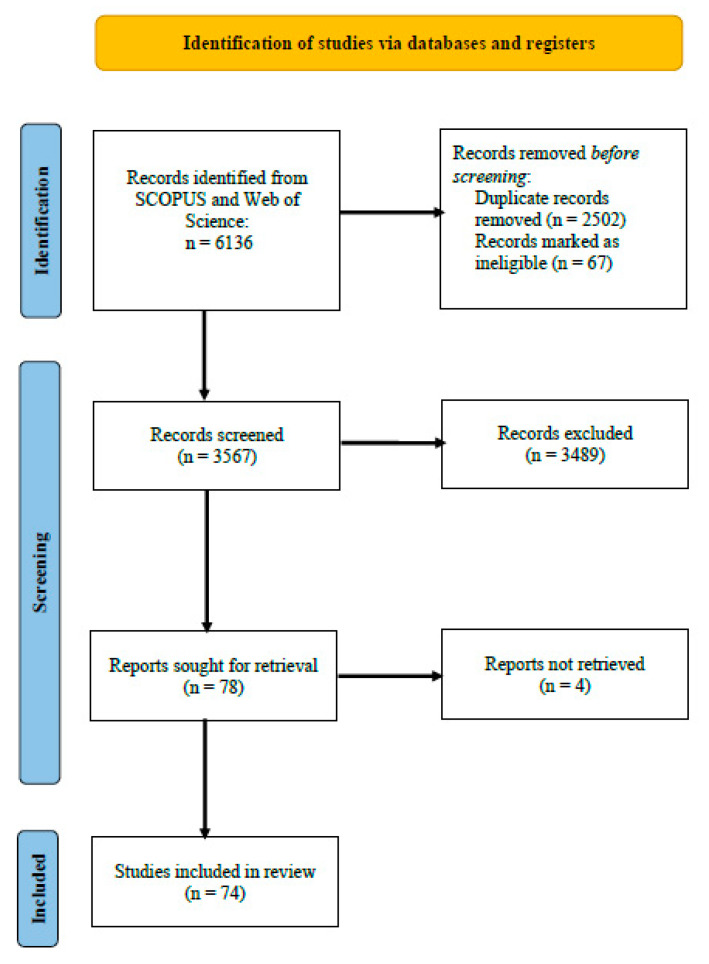
PRISMA flowchart reporting the article selection process for the systematic literature review.

The articles were retrieved from the SCOPUS and Web of Science database and were restricted to the last five years (2017–2021). Keywords such as “plant growth”, “fertilizer”, “bioconversion”, “biofertilizers”, “bioinoculant”, “fungi”, “bacteria”, and “waste” were used to search and retrieve relevant articles from the database into csv format for further analysis. Articles published in English alone were included in the search. Inclusion and exclusion criteria are tabulated in Table 3 and Figure 1. Based on the inclusion and exclusion criteria, screening of articles was carried out after duplicates were removed using MS Excel. The detailed search strategy is presented in Table 1. Thematic analysis of the final list of articles was performed to allocate them into different categories of research trends (Figure 2). A table of evidence (ToE) matrix was constructed from the relevant articles for further inference and interpretation.

## 3. Results

### 3.1. Analysis of Search Results from Scopus and Web of Science

**Figure 3:**
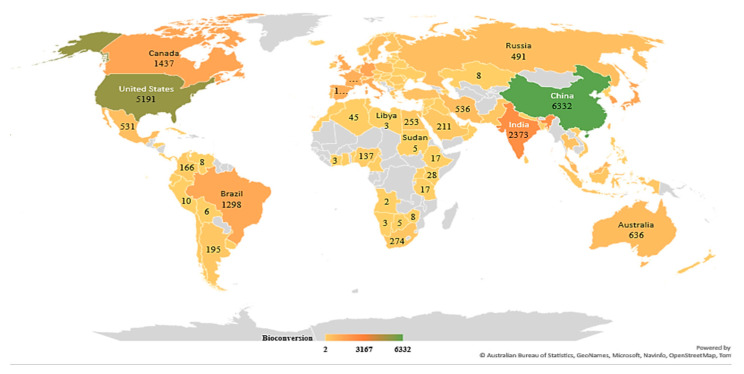
Geographical distribution of bioconversion research during the last five years (2017–2022).

**Figure 4:**
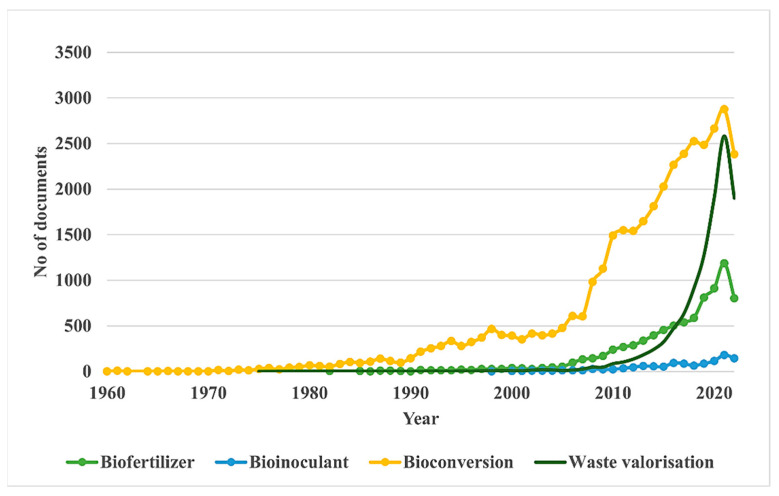
Trends in research publication using the keywords “waste valorisation”, “bioconversion” and “biofertilizer” over time.

Based on the data exported from the Scopus database using a preliminary search of the keyword “biofertilizer” during the years 2017–2022, the geographical distribution of the research was plotted and indicated in Figure 3. As observed on the map, countries such as India, China, and South American countries seem to produce the most research in the field of biofertilizers. A comparison between the number of research articles produced per keyword can be seen in Figure 4.

### 3.2. Bibliometric Analysis by VOSviewer

**Figure 5:**
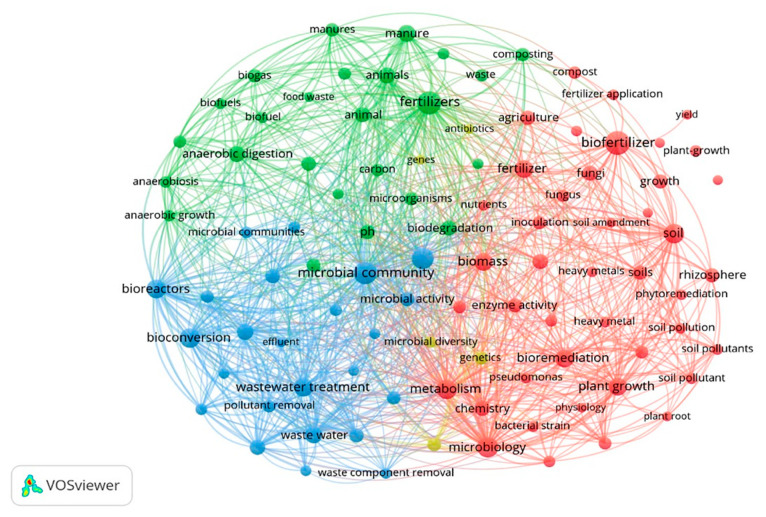
Graphical representation of co-occurrence mapping of relevant keywords associated with fertilizer research.

VOSviewer was used to allocate emerging themes of research and research trends of the past five years through analysis of bibliometric data. Figure 5 represents the co-occurrence mapping of author keywords by the full counting method through overlay visualization. The minimum co-occurrence of the author keywords in the research articles was set to 119 to identify the top 100 major keywords from more than 21,600 author and index keywords retrieved from multiple search strategies. These major keywords present a rather clear picture of the current directions of research in the field of bioorganic fertilizer production. A stark distinction exists between research associated with bioorganic fertilizer production strategies such as composting, anerobic digestion, biodegradation of wastes (manure), plant growth promotion abilities of microbial inoculants (biofertilizers), bioremediation, phytoremediation, and wastewater treatment. The identification of relevant microbial community appears to be at the center of all the connected themes.

**Figure 6:**
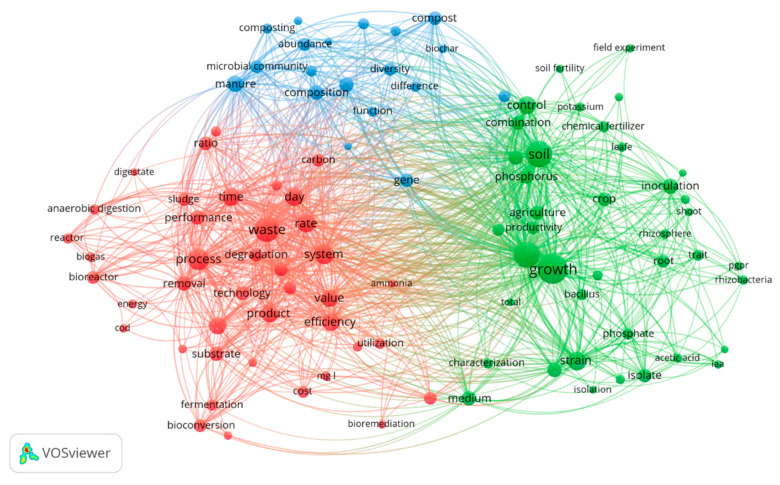
Visualisation of critical terms co-occurrence in textual data used in fertilizer research.

Similarly, Figure 6 presents the plot of the co-occurrence map based on textual data provided in the title and abstracts of the research articles used in the study. Filtering of terms by binary counting, indicating their presence or absence in a research article and setting the minimum number of occurrences to 85, resulted in 100 relevant terms out of 69,247, based on 60% relevance. Co-occurrence mapping of the textual data also revealed three distinct groups of research interest, namely plant growth enhancement, the composition of microbial communities (such as diversity, structure, and succession) in biodigestion processes, and waste treatment strategies. An enormous volume of research has been generated that explores the plant growth promoting the potential of microbial inoculants as biofertilizers based on their ability to produce indole acetic acid (IAA), siderophores, hydrogen cyanide (HCN), ACC deaminase, phytohormone production, salt tolerance, and micronutrient solubilization. Conversion of secondary raw materials to value added products and removal of organic pollutants through waste treatment are some basic waste management techniques often prospected. Similarly, deciphering composition of the microbial communities provides insight into the structure and diversity of the microbial species such as *Proteobacteria*, *Acidobacteria*, *Bacteroidetes*, methanogens, and their significant functional roles in composting and anaerobic digestion processes.

The terms in the three groups could be classified as given in Table 4.

**Figure 7:**
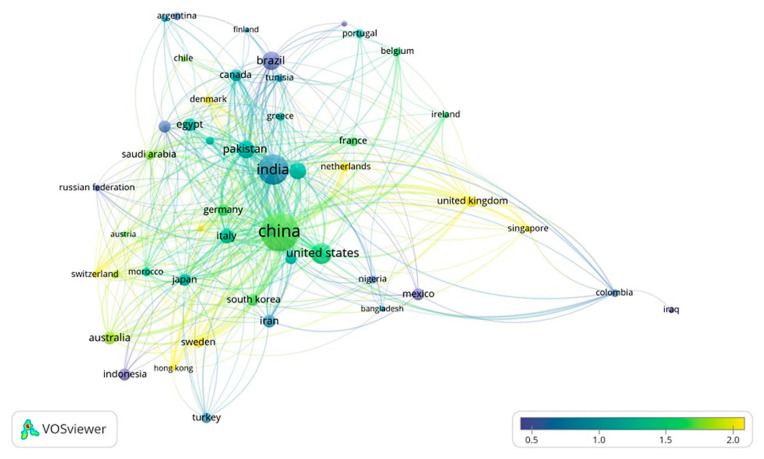
Depicting the co-authorship between countries that engage in intensive fertilizer-based research and their citation weightage.

Figure 7 is a representation of the co-authorship mapping between countries of studies included in this analysis, weighted by total link strength between countries and scored by average normalized citations. Filtered by setting the minimum number of documents as 16 per country, the top 50 countries met the relevant thresholds out of 188. It can be observed that the United Kingdom, Australia, Sweden, Switzerland, Denmark, Netherlands, Singapore and Hong Kong produce some of the heavily cited research in fertilizer research, though not voluminous (yellow in map). However, countries such as India, China, Pakistan and Brazil appear closely associated with co-authoring and collaborating with fertilizer research, producing quite a large number of research articles.

**Figure 8:**
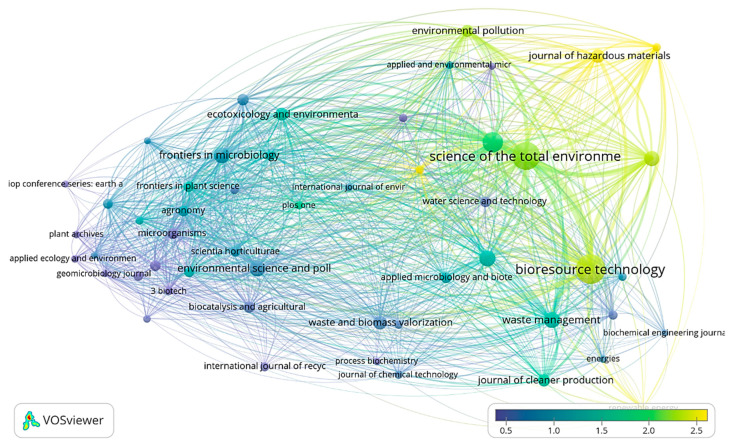
Tracking the journals that are involved in intensive fertilizer-based research and their relative impact factor.

Journals such as Bioresource technology, Journal of Hazardous Materials, Science of Total Environment, and Environmental pollution are some of the most preferred journals and well cited among authors (Figure 8).

### 3.3. Systematic Literature Review

A literature search carried out in the Scopus and Web of Science database using the keywords “biofertilizers”, “waste” and “bacteria” yielded 6136 research articles. After data cleaning of duplicates and ineligible entries, 3567 articles remained. When the exclusion and inclusion criteria were applied to relevant articles based on the title and abstract screening, 3489 were excluded and were unable to retrieve two of them. A final list of 74 research articles was systematically analyzed and a table of evidence (ToE) was constructed and is provided in Table 5 and Table 6

#### 3.3.1. Microbial Inoculants Used for Bioconversion

More than 55% of the studies (*n* = 44) used microbial monoculture for bioconversion of wastes, whereas nearly 20% and 14% (*n* = 16 and 11) used microbial consortia and dual cultures, respectively. Similarly, bacterial cultures were used in nearly 68% (*n* = 58) of the studies, among which five of them used actinomycetes alone and eight of them utilized cyanobacterial biomass for nutrient recovery from wastes. Eighteen studies included purely fungal bioconversion and three studies alone used mixed cultures of fungi with bacteria. *Bacillus* sp. was among the most used bacterial isolates for all types of bioconversions of wastes ranging from dairy wastewater, potash, sewage sludge ash, rock phosphates, bone meal, manure and feather wastes [24,25,26,27,28,29,30]. Secondary to *Bacillus*, cyanobacterial species such as *Scenedesmus, Chlorella,* and *Anabaena,* (*n* = 8) followed by bacterial species *Acidithiobacillus* (n = 3) and fungal species *Aspergillus* sp. (*n* = 7) were used.

Among the consortia used for bioconversion, different types of wastes such as sawdust, chicken litter, wastewater from municipal, food and dairy industry, feather waste from the poultry industry, agricultural residues and petroleum sludge were tried and tested (Table 6). Though studies abound on the plant growth promoting efficiency of *Trichoderma* biofertilizers [31], commercial yet controversial “Effective Microorganisms” (EM) inoculants [32], and microalgal biomass [33], reports on their usage for waste valorisation are not numerous.

The exhaustive list of microbial inoculants used in bioconversion studies in the last five years (2017–2021), the bioconversion strategy adopted in each study, the mode of application of digestate, and the type of plant growth test (plot/pot/germination) have been compiled and presented in Table 6.

However, the identity of the microbial agent (MA-1) mentioned in [52], used for the composting of a combination of wastes consisting of spent coffee grounds, poultry manure and biochar, was undetermined. Furthermore, one of the studies determined the biodegradation of sludge produced by the petroleum industry by the application of Effective Microorganisms (EM) bokashi under anaerobic conditions. The exact microbial composition of EM bokashi used in this study was also undetermined [40].

#### 3.3.2. Bioconversion Raw Materials and Strategies

Three main bioconversion strategies were commonly observed to be adopted in these studies, and they are aerobic composting, solid-state fermentation, and submerged (liquid) fermentation. A team of researchers from Poland utilized carrier-based bioformulation enriched with *Bacillus megaterium* and *Acidithiobacillus ferrooxidans* in granular form to study the phosphate solubilization and plant growth promoting the potential of a waste material composition of wastewater treatment plant (WWTP) ash, also referred to as sewage sludge ash, spent mushroom substrate (SMS) and slaughterhouse waste (bone meal and dried animal blood) [25,30,68,101].

Moreover, all wastewater nutrient recovery and bioconversion-to-biomass studies were undertaken using micro-algal-based biorefinery approaches. Diverse types of wastewaters from sources, such as breweries, toilet (black water), dairy and paddy-soaked rice mill water, were used to cultivate microalgal species such as *Chlorella sorokiniana*, *Scenedesmus obliquus* (ACOI 204/07), *Chlorella* sp., *Dictyosphaerium* sp., *Monoraphidium* sp., *Neochloris* sp., *Scenedesmus* sp., *Chlorella pyrenoidosa*, and *Scenedesmus* sp. (Table 6). Only one study attempted to utilize a cyanobacterial concoction consisting of *Fischerella muscicola, Anabaena variabilis, Aulosira fertilissima* and *Tolypothrix tenuis* for bio-manure production from chili crop waste [37].

A single study reported the use of mesophilic digester (anaerobic digestion) for the production of biofertilizer from a waste composition containing sardine waste, potato peels and poultry waste after the inoculation of the fungus *Aspergillus niger* and yeast *Saccharomyces cerevisiae* [61]. However, in a unique investigation by Feng et al. [53], bioremediation of phthalate acid esters (PAEs)-contaminated soil was achieved by the addition of a bio-organic fertilizer which was synthesized by the inoculation of *Bacillus megaterium* strains in the composting of sewage sludge and agricultural waste.

Seven studies focused on the degradation of keratin-rich chicken feather waste using both fungal and bacterial monocultures of *Alternaria tenuissima*, *Bacillus cereus* PKID1 and *Bacillus pumilus* JYL under submerged fermentation conditions, while ten studies reported the plant growth efficiency of waste digested with phosphate solubilizing microorganisms (Table 3). Biodegradation-based treatment of wastes containing higher ratios of fatty acid residues such as petroleum and kitchen oil were carried out in the presence of EM bokashi consortia and *Pseudomonas aeruginosa* PA-3 [40,71].

*Corynebacterium glutamicum,* a Gram-positive bacterium mostly used in the industrial production of amino acids, has been applied by a research team at the Federal University of Parana in Brazil for the fermentation-based production of biofertilizers from sugarcane molasses [57,87].

#### 3.3.3. Plant Growth Tests and Mode of Application

On the other hand, nearly 50% of the studies adopted pot (*n* = 38) and plot tests (*n* = 25) to determine the efficiency of their biofertilizer formulation. Only three studies determined their plant growth promoting the potential of their product using seedling germination tests. More than 70% of the studies (*n* = 55) employed their product as a soil amendment for their plant growth tests.

Foliar application was tested in three studies where the fermentate obtained from sugarcane molasses by *Corynebacterium glutamicum* was tested on potato and *Brassica campestris* var. Pekinensis and lignin waste fermented by phosphate solubilizing mixed culture of fungal strain *Meyerozyma guilliermondii* and bacterial isolate *Providencia rettgeri* was tested on cowpea plants [57,87,97]. Soil irrigation using the fermented wastes of dairy wastewater and kitchen oil waste was applied on cabbage and mung bean plants in plot tests. Feng et al. [53] employed a modified strategy of soil amendment by spiking the pot tests with pollutants at appropriate concentrations before plant growth tests.

## 4. Discussion

Bioconversion studies are being conducted at much higher volumes than biofertilizer research, whereas waste valorization studies have gained momentum only in the last decade (Figure 2). However, the volume of biofertilizer-associated research is comparatively lower compared to waste valorization and bioconversion research. Moreover, the graph indicates that biofertilizer and waste valorization research have been comparatively recent when compared to bioconversion studies. It can also be observed that there is a wide gap between the research focussing on “plant growth” promoting microorganisms and their application for waste bioconversion studies.

During the systematic literature review, the authors observed that the predominant themes among the search results were associated with anaerobic digestion (AD), vermicomposting, algal nutrient recovery from wastewater, and biofertilizer. It seems necessary to highlight the relevance of anaerobic digestion (AD), composting (including vermicomposting), and nutrient recovery studies from wastewater treatment plants (WWTP) in bioconversion of waste biomass. Other minor themes that were explored in literature survey were production technology (pre-treatment techniques) of bio-based fertilizers, testing of the impact of microbial biofertilizers (bioinoculants and biostimulants) in the specific host plant, carriers used for biofertilizer formulation and study of microbial communities after application of biofertilizers.

### 4.1. Current Strategies of Fertilizer Production Using Bioconversion

#### 4.1.1. Composting and Vermicomposting

Composting is essentially a traditional system used for the aerobic and thermophilic digestion of organic waste such as leaf litter and cow dung, converting both solid and liquid waste to humus which is essential for plant growth. Several strategies such as rotary drum, windrow, and aerated static piling where *n* number of factors can be adjusted such as type of substrate, substrate capacity, duration of composting, and quality of end-product [102]. Composting by the employment of earthworms (*Eisenia foetida, Eudrilus eugeniae, and Perionyx excavatus*), vermicomposting, also enhanced the quality of composted organic wastes. Vermicomposting has been noted to enhance the nutrient quality of the compost, as well as removal of potential pollutants through the aid of the invertebrate’s digestive system. [103]. However, the system comes with its own set of disadvantages, such as detection of pathogens in the compost, low micronutrient and humus content, long duration of the complete process and considerable generation of odour. In addition to design of appropriate composter, supplementing the compost waste materials with functional microbial inoculum to increase the compost quality has also been assessed in several studies [104].

In a study by [105] wood-rot fungi strains *Lasiodiplodia* sp. F1, *Verticillium* sp., and *Trametes versicolor* were observed to remove Tiamulin, a common antibiotic found in compost, from swine manure. A strain of *Thermus* sp. was isolated from pharmaceutical sludge with the potential for biodegradation of antibiotic ciprofloxacin from swine manure compost, where it is a common contaminant [106]. Bioaugmentation of functional microbes belonging to two different groups (PGPB and plant pathogen-suppressing bacteria) to a composting of spent coffee grounds (SCGs), rice bran, and biochar produced a significant increase in germination index and reduction in disease incidence [89]. Similarly, composting of palm oil empty bunches and sugarcane biomass in the presence of lignocellulolytic consortia of bacterial and fungal strains resulted in a decrease of recalcitrant cellulose and lignin content, as well as a C:N ratio [107].

Succession of different microbial communities has also been observed to be correlated to the composition of waste materials in the compost. Nakasaki et al. [108] identified the dominance of *Bacillales* taxa throughout the composting of organic-only waste, and the presence of Symbiobacterium at the last phase of composting. When composting of swine manure was amended with coconut shell biochar, the dynamics of the bacterial community diverged to include keratin degrading *Firmicutes* and *Actinobacteria*, to the already dominant *Bacilli* species [109]. This hints at the premise that supplementing substrate-specific microbial inoculum can enhance compost quality, nutrient content, and plant growth as a result.

#### 4.1.2. Anaerobic Co-Digestion (AcoD) of Waste

Anaerobic digestion is one of the key mechanisms through which organic matter (or wastes) is converted to fuels such as biogas and its digestates as organic fertilizers. The digestates have exhibited quite an agronomic potential [110]. AD digestates have been produced from different types of biomasses and tested across diverse types of crops, wherein they have been proven to affect a significant increase in plant growth. Jamison et al. [101] tested the efficacy of a combination of digestates from two wastes such as food waste, and lignocellulosic biomass on the growth of *Brassica juncea* (Kai Choy). It was found to significantly increase the nitrogen use efficiency of plants, plant biomass, and root length. However, digestates from olive mill wastewater, domestic wastewater, and cow dung were found to induce phytotoxicity [111]. A similar study by [112] used combinations of cattle manure, cheese whey, and pig manure to determine their agronomic suitability. The study revealed that the digestate had appropriate concentrations of NPK to be utilized as fertilizer but was unstable for long-term storage and carried fecal pathogens that can be transmitted to the soil. The study also recommended a suitable post-treatment of the digestates prior to field application.

The process of anaerobic digestion often involves the addition of a seed microbial inoculum that is dependent on the type of waste (or substrate) that needs to be digested, and the inherent microbial community that drives the whole process such as methanogens and acetogens. Most often, the inoculum is prepared from an already existing digester depending on the physicochemical factors that determine AD such as pH, temperature, C:N ratio, volatile fatty acids (VFA), and others. When anaerobic rumen fungi were added to anaerobic digestion of cattle manure, it was found to increase the biogas yield [113]. Similarly, the addition of livestock manure to anaerobic co-digestion of palm oil mill effluent and brewer’s spent grain was found to enhance the plant growth promoting bacteria (PGPB) community and micronutrient levels (K, P, Ca, Mg, S, N). While pretreatment of biomass is often necessitated, recent research has been propagated to turn toward microbial pretreatment for enhancement of the AD process.

Among the AD studies, the primary focus of the research was on the prevalence of ARGs, coliform, pathogens, different types of waste digestion, and life cycle assessment (LCA), including several exhaustive reviews on AD and composting. For instance, Golovko et al. [114] estimated the risk associated with the widespread use of anaerobic digestates from biogas plants as fertilizers by characterizing the number of heavy metals, food-borne pathogens, antibiotic-resistant bacteria, antibiotics, pesticides, and other chemicals of emerging concern (CECs). A similar study by Södergren et al. [115] assessed the microbiological safety of using the liquid digestate produced from the biogas plant for the hydroponic cultivation of vegetables.

#### 4.1.3. Nutrient Recovery and Wastewater Treatment by Microalgae

An important sustainable technology with considerable contribution to the circular economy is converting wastewater streams from various sources such as municipal wastewater and industrial effluents to recover nutrients with application as fertilisers. Microalgae, also known as cyanobacteria, are photoautotrophic microorganisms that are capable of fixing carbon dioxide to organic biomass. They are resilient to grow in diverse environments and can generate large amounts of biomass rich in physicochemical composition of lipids and proteins from using the nutrients and micronutrients available in the wastewaters. The available technology including the design of the photobioreactor, downstream processing, large-scale implementation and its economic feasibility has been extensively discussed by [116,117].

To add more value to the end-products, an algal consortium consisting of *Chlorella* sp. and *Scenedesmus* sp. grown in wastewater was used for lipid production. When the residual deoiled biomass was supplemented as organic fertilizers for the growth of *Solanum lycopersicum*, it was found to enhance plant biomass and tomato yield [91]. Similarly, deoiled biomass of a monoculture of *Scenedesmus* sp. provided 100% nitrogen required for rice crop, and significantly increased grain weight, panicle weight, and plant dry weight [79]. Microalgae is a powerful tool for the bioconversion of wastewater into biofertilizers and fits tightly into the biorefinery approach [118,119].

#### 4.1.4. Bio-Organic Fertilizers

Several studies reveal that treatment of soil or plant growth with co-inoculation of microbial fertilizer and soil amendments enhances the availability of NPK, micro-, and macronutrients to the plants. The use of these inoculants for the targeted digestion of waste biomass for the elution of NPK and other plant-growth promoting factors, and its application as a “bioorganic fertilizer”, is a considerable bioconversion technology. Depending on the biochemical composition of the waste biomass, a suitable microbial inoculant or consortia can be applied for efficient bioconversion or solubilization. Microbial inoculants used in the digestion of substrates and tested for its capacity to promote plant growth have been enlisted in Table 3. Meanwhile, the trends in the research are predominantly associated with the application of microbial biofertilizers, which can be classified into five main types, and have been extensively reviewed by several authors:

- plant growth promoting rhizobacteria or bacteria and fungi (PGPR, PGPB and PGPF) [120,121,122];
- phosphate and potassium solubilizing bacteria (PSB and KSB) [123,124];
- mycorrhizal fungi (AMF) [125];
- Nitrogen-fixing bacteria (NFB) [126,127];
- Non-mycorrhizal fungi and endophytic fungi [122,128].

### 4.2. Mechanism of Microbial Bioconversion

#### 4.2.1. Microbial Fermentation

It is well-understood that microorganisms require a constant supply of carbon and nitrogen feedstock for their survival, and possess a large array of tools such as biochemical pathways, production of organic acids, and enzymes to utilise them. Agro-industrial wastes, and other types of wastes, make an appropriate nutrient medium for microbial fermentation. The term microbial “fermentation” indicates anaerobic catabolism of diverse substrates (e.g., sugars) to produce ATP as their source of energy. The catabolism is not complete oxidation of substrate to carbon dioxide, but rather partial breakdown to organic acids such as lactic acid, acetic acid and ethanol [129]. Agro-industrial wastes and municipal solid wastes are often rich sources of micronutrients (NPK) and protein content that enhance plant growth in nutrient-deficient environments.

While microbial fermentation in bioreactors is employed by several industries in food, and pharmaceutical industries for the production of metabolites, the mode of fermentation can be classified into two: submerged fermentation (SmF) and solid-state fermentation (SSF). Submerged fermentation indicates the process of microbial catabolism of substrates in the presence of water or water-based nutrient media, mostly in bioreactors where parameters such as pH and aeration are controlled [130]. Whereas solid-state fermentation, as the name indicates, is carried out using substrates which require or have low water content, produce high concentration of products, and have simpler downstream processing. Both methods have their advantages and pitfalls in their use for biomass conversion, which are discussed extensively by [131]. Buntic et al. [132] compares these two modes for the fermentation efficiency of cellulose production from waste tobacco using *Sinorhizobium meliloti* strain 224 as an inoculum. Several factors such as the type of biomass (lignocellulosic, MSW), the type of strain (PGPB, PSB, AMF), base of bioformulation (liquid, solid), and the type of scaling-up must be optimised to choose the suitable mode of fermentation [133].

#### 4.2.2. Microbial Lignocellulolysis, Keratinolysis, and Chitinolysis

Agroindustrial and seafood wastes are rich sources of cellulose, lignin, hemicellulose, chitin, and pectin which are recalcitrant polysaccharides that can be decomposed into pentoses and hexoses, which are nutrients for microorganisms. Microorganisms are bio-factories that produce an array of cell wall degrading enzymes (CDWE) such as glycosidase, amidase, β-glucosidases, ligninases (laccases, Mn-peroxidases, polyphenol-oxidases), pectinases, xylanases, mannanases, cellulolytic oxidases, and cellulases [134,135,136].

Green waste fraction of MSW composted using ligninolytic and cellulolytic fungi was amended to soil for the growth of red chillies and found to enhance plant biomass and its chlorophyll content [67]. Fruit and vegetable wastes (FVW) co-digested in a two-phased manner with slaughterhouse wastewater (SWW) in the presence of *Trichoderma reesei* revealed reduction in its lignin and cellulose content, and high C:N ratio required in biofertilizers [137]. Palm oil empty bunches and chopped sugarcane biomass with a high ratio of lignin and cellulose were heavily reduced upon the inoculation and composting in the presence of up to 4% cellulolytic and ligninolytic bacteria and fungi [107]. Sweet sorghum bagasse (SSB) was subjected to fungal pretreatment by *Coriolus versicolor* through solid-state fermentation and was found to be an efficient pretreatment method which made the substrates more vulnerable and accessible to prolonged enzymatic saccharification [138]. Biotransformation of lignocellulosic biomass (LCB) and low-density oxodegradable polyethylene (LDPEoxo) through co-digestion using *Pleurotus ostreatus* as inoculum, producing a “bio-organic” (BoF) fertilizer that enhanced plant growth of *Allium cepa*. It was correlated to the synergistic effect of hydrolytic enzymes and oxidative enzymes (ligninases, cellulases and hemicellulases), via co-metabolism [139]. Silva et al. [140] provides a detailed insight on how CDWE can be manipulated through enzyme engineering to encompass lignocellulosic biomass into the biorefinery concept.

Similar to agro-industrial wastes, seafood processing wastes such as crustacean shell waste and shrimp waste contain high ratios of chitin, a polysaccharide with nitrogen functional group, micronutrients in the form of calcium carbonates, phosphates and proteins. Microbial bioconversion of shrimp and crab shell wastes was attempted using a chitinase-producing *Alcaligenes faecalis* SK10 strain and assessed for its potential as fertiliser on the growth of growth of *Pisum sativum* and *Cicer arietinum*. When compared to fertiliser doses of raw chitin, the fermented hydrolysate showed performances on par for several parameters such as micronutrient content (NPK) of soil, plant growth, and chlorophyll content [82]. A marine bacterium *Exiguobacterium antarcticum* DW2 was found to produce a cold-temperature chitinase that hydrolyzes chitin to bioactive polysaccharides [141]. A chitinase gene was identified in the biosynthetic gene cluster of an endophytic *Bacillus subtilis* Dcl1 that isolated from the dried rhizome of *Curcuma longa,* which also contained several plant promoting factors such as drought tolerance and IAA production [142]. Strains of chitinolytic *Streptomyces* were isolated from lobster processing wastes exhibited demineralization, and deproteinization of lobster shells in addition to inducing disease resistance against *Botrytis cinerea* in *Arabidopsis* [66]. Moreover, these microbial strains that produce chitinase have an advantage of carrying host–defense systems for plant growth against pests [143].

On the other hand, feather waste from the poultry processing industry is one of the richest sources of protein, available in the form of keratin. However, due to the recalcitrant and insoluble structure of keratin, microbial isolates produce specialised serine metalloproteases that enable complete catabolism. *Kocuria rhizophila* p3-3 isolated from chicken was utilized for the valorisation of chicken feathers, and degraded them up to 52% in just 4 days at room temperature [48]. Strains *Trichoderma asperellum* and *Trichoderma atroviride* were cultivated on chicken feathers and sheep wool produced microbial formulations that had plant biostimulant properties and contained protein hydrolysates and metabolites that impacted seed germination, along with crop productivity [144]. Tamreihao et al. [145] provides a detailed overview of the agronomic potential of feather valorization and feather-derived keratin hydrolysates in the adoption of circular economy.

#### 4.2.3. Microbial Micronutrient Solubilization—Phosphorus

Phosphorus is an essential micronutrient for plant growth, and natural resources for phosphorus fertiliser production currently used are two types of raw materials called sedimentary rock (phosphorite) and volcanic sediment (apatite). However, there is a need to incorporate the concept of circular economy and biorefinery into production of NPK fertilizers, as we might reach peak phosphorus soon [146]. Several types of wastes including agricultural residues and wastewater sludge contain different forms of phosphorus which are often in unavailable forms to plants. In addition, recovery of these phosphates for application in plant growth through industrial means can be expensive and defeats the purpose of a circular economy.

However, phosphate solubilizing microorganisms possess the ability to enhance the release of phosphates from wastes through production of acids such as oxalic acids, and enzymes such as phosphatases and phytases. A study by [147] tested the efficiency of *Bacillus megaterium, Bacillus subtilis,* and *Bacillus cereus* isolates to solubilise phosphates from three different waste materials (poultry bones, fish bones and ash). Production of acetic, gluconic, propionic, lactic and succinic acids were revealed to play important roles in phosphate solubilization, and fish bones provided the highest concentration of released phosphates. In a similar study, a thermotolerant consortium of *Streptomyces* was added as inoculum to the composting of organic wastes and was noted to solubilize up to 50 µg/mL from the wastes [148]. When a consortium of four strains of *Penicillium bilaiae, Penicillium aculeatum* and *Aspergillus niger* were added to a combination of sewage sludge ashes and biochar, it was observed to increase the P availability in spring wheat [86].

More than 325 mg/L of phosphate was solubilized from hydroxyapatite produced from *Coptodon rendalli* (tilapia) fish scales by the strain *Acidovorax oryzae* ZS 1–7 [149]. Production of acid and alkaline phosphatase enzymes led to the increase in acid levels and available P when *Herbaspirillum seropedicae* was inoculated into a compost made up of poultry litter and crushed grass (*Pennisetum purpureum* Schumach) [150]. Compost made up of by-products of sugarcane such as filter cake and ash, inoculated with phosphate solubilising *Pseudomonas aeruginosa* PSBR12 and *Bacillus* sp. BACBR01 improved the supply of available P to 150 kg ha^−1^, increased sugarcane yield, acid phosphatase and β-glucosidase activity [73]. The use of phosphatases, phytases, and phosphate solubilizing microorganisms to solubilize unavailable P from agroindustrial wastes can reduce our dependence on finite P resources [83,151].

Similar to P, other micronutrient solubilizing microbes (MSMs) such as zinc (Zn), nickel (Ni), manganese (Mn), magnesium (Mg), potassium (K), copper (Cu) and selenium (Se) have been exploited as microbial biofertilizers and carry applicable advantages as “bio-organic” fertilizers for mineral-deficient soils and waste valorisation, synergistically [152,153].

#### 4.2.4. EPS Production

Bacteria are usually found as communities attached to a surface rather than as planktonic cells floating in a nutrient medium and secrete an exopolymeric matrix that contains proteins, nucleic acids, and exopolysaccharides (EPS), which is often visible as a slimy layer. Seneviratne et al. [154] observed that, when fungal strains were incorporated with N_2_-fixing rhizobial strains, they formed fungal–rhizobial biofilms (FRBs) that had potential in biocontrol and fertilisation of nitrogen deficient soils. Rathnathilaka et al. [155] determined that supplementation of BFBF to conventional large scale paddy cultivation increased crop yield, soil micronutrients, and influenced microbial communities. Similarly, bacteria can also be utilised to produce exopolysaccharides (EPS), a major component of biofilms from wastes. *Halomonas* strains were used to produce EPS rich in galactose from cheese whey wastes [156]. EPS production was evaluated by inoculation of lactic acid bacteria (LAB) *Leuconostoc mesenteroides* WiKim32 in spent media wastewater (SMW) derived from kimchi fermentation and exhibited antioxidant activity [157]. Solid-state fermentation of cane bagasse and broadbean seed capsule by *Kosakonia cowanii* LT-1 yield maximum yield of EPS, promoted germination index, and growth vigour in *Zea mays* [55]. Exopolysaccharides and biofilm biofertilizer (BFBF) produced by plant growth promoting bacteria (PGPB) have the potential to act as a biocontrol agent and protect plants against abiotic stress such as heavy metal contamination or drought [158].

#### 4.2.5. PAE and LCT Biodegradation

Phthalate esters (PAEs) are contaminants of emerging concern (CEC) in agricultural soils of several countries and are quite often correlated with improper garbage disposal resulting in the leaching of Di(2-ethylhexyl) phthalate (DEHP), Di-n-butyl phthalate (DnBP) and Diisobutyl phthalate (DiBP) into water sources of irrigation [153,159,160,161,162,163,164]. Feng et al. [53] adopted the incorporation of a “bio-organic” fertilizer strategy to address this concern. A mixture of sewage sludge and agroindustrial residues was concocted with the addition of two bacterial strains of *B. megaterium* (YJB3, YLYP1) that had PAE-degrading as well as phosphate solubilizing abilities. A significant decrease in translocated concentration of PAEs in plants (chinese flowering cabbage) cultivated in contaminated soils and an increase in crop yield was observed. Similarly, application of organic fertilizer to plastic-shed soil was found to significantly enhance *Planifilum, Geobacillus,* and *Thermaerobacter* species, and correlated with the degradation of oligomeric dibutyl phthalate (DBP) to monomeric monohexyl phthalate (MEHP) and mono-n-butyl ester (MBP) [162].

There has been extensive research in the biodegradation of hydrocarbons including oil wastes generated from the food industry [160,161]. However, the potential of waste kitchen oil, having higher ratios of long chain triglycerides (LCT), as an ideal feedstock for biodiesel production through enzymatic transesterification, has been much explored, Marchetti et al. [159] identifies that the efficiency of kitchen waste oil degradation is enhanced by the co-digestion of pig slurry in anaerobic digestion. However, in a unique study, the bacterial strain *Pseudomonas aeruginosa* PA-3 was used to break down kitchen waste oil into shorter chain fatty acids, and the by-products were revealed to enhance plant growth and biomass in cabbage [71].

### 4.3. Microbial Community Analysis

The advent of culture-independent techniques, such as high throughput sequencing and metagenomics, has been used to understand the dynamics of the microbial communities in response to the concentration of different substrates in the available waste streams.

Microbial community analysis using amplicon sequencing and metagenomic approaches was also quite common, especially in anammox-associated studies [165,166]. A consistent amount of research was also focused on the remediation of metal-contaminated soils using microbial strains, plants (phytoremediation), or a combination of both. High-throughput sequencing was employed to determine the changes in the microbial community prior to and post vegetation on zinc (Zn) smelting waste slag [167]. Similarly, the effect of inoculating *Streptomyces griseorubens* JSD-1 on the co-composting of rice straw and swine manure was found to significantly change the microbial biomarkers to the *Bacteroidetes* genera, which positively correlated with an increase in plant-available micronutrients NPK [168]. In a significant study by Jiang et al. [169], high-throughput amplicon sequencing of the 16S rRNA gene isolated from the co-digested pig manure and food wastes revealed that a suitable inoculum can drive the microbial dynamics and community in favour or against the digestion more than the physicochemical composition of the waste substrate.

### 4.4. Nomenclature

It is also important to note that several authors referred to the anaerobic digestates as “biofertilizers”, though the standard definition of the term “biofertilizers’’ often denotes the use of biologically active preparations that contain beneficial microorganisms that enhance plant growth and yield. Similar nomenclature can be seen in [170,171,172,173] and many more. Similarly, the term “organic fertilizer” is often used to indicate the production of a sustainable fertilizer from by-products of agriculture, and pastoral farming, such as manure, litter, and poultry droppings by means of composting or anaerobic digestion [174]. However, due to the growing understanding of the microbial community’s role in converting waste into plant available nutrients, a grey area arises in the usage of varying terminology. Since the liquid or solid digestates from anaerobic digesters or composters often contain a diversity of microorganisms in addition to organic, plant essential nutrients, the question of it being a “biofertilizer” or “organic fertilizer” is debatable. However, this necessitates the reinstatement of proper terminology for microbial inoculants used for the sole purpose of plant growth, such as “microbial biofertilizer.

It is necessary to highlight the significance of nomenclature applied in a significant number of research papers evaluated in this study. Though the term “biofertilizer” has been traditionally associated with the application of microbial inoculum or formulation for enhancing plant growth and yield, it has been observed that the term has also been applied to indicate “bio-based” or organic fertilizers. As per the literature, it is observed that fertilizers made from composting, AD, manure, and other waste digestates are also termed biofertilizers when the correct terminology indicates the use of organic fertilizers or bio-organic fertilizers [34]. Here, the scenario indicates a lack of awareness of term differentiation between microbial biofertilizers, denoting the use of fungi, protozoa, archaea, and bacteria for plant growth promotion, biofertilizers that indicate the use of other living organisms for plant growth, followed by organic or bio-organic fertilizers that stipulate the application of manure, compost or other waste-based plant growth promoting material in agriculture.

Here, the authors would like to highlight four different fertilizer products associated with the terminology “biofertilizers” along with their mechanisms

(a) Microbial fertilizers such as bio-inoculation of plant-growth-promoting bacteria (PGPB) and arbuscular mycorrhiza (AM), biocontrol microorganisms (bioorganic fertilizers);
(b) Organic fertilizers which are made from composting, AD, manure, and other waste digestates;
(c) Biofertilizers made from agro-industrial, organic wastes (BFW) by the application of phosphate- and potassium- solubilizing, keratinase-digested, bacteria and fungi;
(d) Combination biofertilizers.

It can be summarised that production strategies of biofertilizers have been approached in three different modes, microbial biofertilizers, biofertilizers from waste processed by composting or anaerobic digestion, and biofertilizers produced from wastes by the effect of microorganisms through bioconversion. Lacunae in other aspects of biofertilizer research such as carriers used for inoculant stability and different modes of inoculation like seed immersion, root inoculation, and foliar spray have also been noted. The usage of digestates and processed wastes from anaerobic digestion (AD) and composting as organic fertilizers, often wrongly termed biofertilizers, are extensively studied in comparison to microbial inoculant studies [175,176,177].

### 4.5. Mode of Valorized Biomass Supplementation

Various modes of fertilizer application have been explored by several researchers, and co-inoculation of microorganisms along with valorised biomass appears to be a predominant strategy. In contrast to the biorefinery approach of valorizing waste through the addition of substrate-specific microbial inoculum, microbial strains or consortia were supplemented along with organic fertilizers. The performance of biochar derived from the pyrolysis of various types of agroindustrial wastes have been tested and identified as a reliable carrier for the microbial inoculants. The viability of microbial inoculants, their concentrations, storage stability and shelf life are some of the common factors often studied together [178,179].

Apart from the co-inoculation approach, protein hydrolysates derived from the microbial degradation of chicken feathers was tested as foliar spray for its plant growth ability on vegetable crops [60]. While different modes of fertilizer supplementation have been for microbial biofertilizers and liquid digestates from anaerobic digestion or composting, it has not been evaluated extensively for liquid fertilizers from microbially valorised wastes. Soil amendment or irrigation of roots seem to be the most preferred or researched mode of supplementation. More research is needed to identify the impact of mode of supplementation on crop yield and pest control.

Though studies that utilize microorganisms with diverse enzymatic and solubility properties have been performed, very few have validated the potential of their digestate or fermented products through plant growth tests. Identifying the prospect of a digestate to act as a “bio-organic” fertilizer by their composition of NPK can be considered insufficient, as the availability of the NPK to plants cannot be guaranteed. Similarly, many studies have performed pot- or plot-based random block design-based experiments using combinations of microbial inoculum along with waste biomass.

### 4.6. Lacunae in Biofertilizer Research

While the isolation and characterization of plant growth promoting bacteria and fungi were numerous, their application for bioconversion of wastes into digestates and biofertilizers was comparatively low. Moreover, functional gene studies that linked an appropriate molecular marker to certain plant growth-promoting factors such as phosphate solubilization, heavy metal accumulation, ligninocellulose-, chitinolytic-, and keratinolytic- gene clusters were also not abundant. Similarly, most of the pot and plant-based plant growth studies were conducted in natural conditions or soil systems that mimicked natural conditions. Studies that focussed on the application of biofertilizers or microbial inoculants on contaminated soils were comparatively lower. While many studies tested the bioformulation variants of their microbial inoculant using peat and biochar as carriers, it is unknown from the available documentation whether an appropriate risk assessment was conducted before its deliberate environmental release. Especially in plot tests of digested wastes containing microbial inoculants, it seems necessary to adopt quality assessment and antibiotic resistance testing of the formulation, which is restricted to AD studies of sewage sludge and associated wastes.

## 5. Conclusions

While this study is by no means exhaustive, it is a timely indicator that the production of sustainable biofertilizers by the valorization of waste through the application of a varied spectrum of microorganisms is a successful, yet less pursued strategy. Preference for algal nutrient recovery from wastewater streams has been observed among researchers in waste valorization, as well as restrictive to agro-economical countries such as India and China.

## Figures and Tables

**Table 1:** Search strategy used to retrieve relevant research articles.

Search Strategy	Scopus	Web of Science
biofertilizer * AND waste * AND fung *	56	64
biofertilizer * AND waste * AND bacteria *	109	121
“Plant growth” AND waste * AND bacteria *	449	461
“Plant growth” AND waste * AND fung *	202	217
**fertilizer AND waste AND fungi**	148	154
**fertilizer AND waste AND bacteria**	632	412
**biofertilizer AND waste**	298	252
**biofertilizer AND bacteria**	606	507
**biofertilizer AND fungi**	268	252
**bioconversion AND waste AND fungi**	127	99
**bioconversion AND waste AND bacteria**	469	203
bioinoculant * AND waste *	11	19
**Year range was limited to 2017–2021 (last five years)**		

“*” indicates the truncated version of the term (fung * represents fungus, fungi, fungal) as recognised by the search algorithm.

**Table 2:** PICO framework utilization for the development of the research question.

Problem (P)	Extensive Use of Chemical Fertilizers
Intervention (I)	application of biofertilizers produced by the waste valorization
Comparison (C)	different types of microbial inoculants used for waste valorization for biofertilizer production
Outcome (O)	Identification of microbial inoculants with potential and used for wastes bioconversion strategies

**Table 3:** Inclusion and exclusion criteria used in this study.

Inclusion Criteria	Exclusion Criteria
Studies that used plant growth studies in pot, or plot settings	Review articles associated with anaerobic digestion or composting
Articles published during 2017–2021	Studies that were related to waste pre-treatment techniques, biorefinery, microalgae production
	Studies that studied microbial inoculants for the bioconversion of wastes into fertilizers only on plate-based or culture-based setups without plant growth assessments
Studies that assessed microbial inoculants for the bioconversion of wastes into fertilizers	Conference proceedings and posters were excluded

**Table 4:** Some of the terms allocated into clusters based on mapping of textual data.

Parameters of Interest in Fertilizer Studies	Plant Growth Promoting Microorganisms	Keywords in Circular Economy	Parameters of Plant Growth Promotion by Microorganisms
Ash, soil amendment, Biofertilizer treatment, Carrier material, seed inoculation, co-inoculation, chlorophyll content, available p, bacterial community, abundance, field experiment, root, shoot, dry and fresh weight, urea, greenhouse experiment, crop yield, harvest, growth parameters, NPK, nutrient content and uptake, *Oryza sativa*, *Triticum aestivum*, soybean	*Bacillus subtilis*, *Burkholderia, Fusarium oxysporum, Enterobacter, Azotobacter chroococcum, Bacillus megaterium,* endophytic bacterium, solubilizing bacteria, *Trichoderma, Pseudomonas, Rhizobium,* rhizobacteria, *Serratia*	Agricultural waste, anaerobic digestion, biochar, biofuel, biogas, composting, circular economy, feedstock, digestate, electricity, energy, environmental impact, recovery, recycling, reduction, industry, pollution, sustainable development, circular economy, waste valorization, wastewater, food waste, sewage sludge	ACC deaminase, acetic acid, ammonia, biocontrol, biopesticide, catalase, protease, chitinase, IAA production, nitrogen fixation, phosphate solubilization, phytohormone, salt stress, seedling germination, siderophore and gibberellic acid production

**Table 5:** Different types of consortia used for bioconversion of wastes.

Inoculum Type	Type of Microorganism	Microbial Inoculant Used	Waste Used	Reference
**consortia**	actinomycetes	Actinomycetes	sawdust plus chicken litter	[34]
**consortia**	bacteria	*Aeromonas, Bacillus, Thaueraamino aromatica, Acinetobacter* (a consortium of 7 isolates)	Dairy Wastewater	[24]
**consortia**	bacteria	cellulolytic bacterial consortium	organic liquid waste (rice washing, coconut water liquid, tofu liquid waste, and palm oil mill liquid waste)	[35]
**consortia**	bacteria	consortium of decomposer microorganisms: *Bacillus pumilus AL16, Microbacterium terregens* AC1180, *Aeromonas* sp. B5376, *Arthrobacter globiformis* AC1529, *Streptomyces olivocinereus* AC1169 and *Acinetobacter* sp. B390.	feather-downy waste and dung	[28]
**consortia**	bacteria	lactic acid bacteria	silage (BE) of shrimp head, molasses, and milk	[36]
**consortia**	cyanobacteria	cyanobacteria (*Fischerella muscicola, Anabaena variabilis, Aulosira fertilissima, Tolypothrix tenuis*)	Chilli waste (stems and leaves after harvesting the fruit)	[37]
**consortia**	cyanobacteria	*Chlorella* sp., *Dictyosphaerium* sp., *Monoraphidium* sp., *Neochloris* sp. and *Scenedesmus* sp.	municipal wastewater (MWW) with simulated flue gas	[38]
**consortia**	mixed	*Enhydrobacter aerosaccus* (ACCA2 JX042472), *Aspergillus* sp. (ALUH KT356201)	raw rice husk (RRH)	[39]
**consortia**	mixed	*Acidithiobacillus*, *Beijerinckia indica* and *Cunninghamella elegans*	rock biofertilizers mixed with sulfur	[27]
**consortia**	unknown	EM bokashi	petroleum sludge	[40]

**Table 6:** Table of evidence (ToE) matrix constructed based on systematic literature review of 74 research articles.

Waste Used	Bioconversion Strategy	Microbial Inoculant Used	Plant Tested	Mode of Application	Type of Test (In Vitro/Pot/Plot)	Role of Microbe	References
**Cattle hooves (slaughterhouse waste)**	submerged fermentation	*Lichtheimia corymbifera* AS1	*Vigna mungo*	irrigation with hydrolysed hooves	germination and pot tests	keratinolysis	[41]
**Olive mill solid waste (OMSW)**	fermentation	*Aspergillus tamarii*	*Vicia faba*	soil amendment	germination and pot tests	fermentation	[42]
**petroleum sludge**	biodegradation	EM bokashi	Onion	soil amendment	pot tests	biodegradation	[40]
**Municipal solid wastes (MSW)**	composting	*Streptomyces* sp. Al-Dhabi 30	*Solanum lycopersicum*	soil amendment	germination and pot tests	plant growth promotion (IAA, siderophore, phosphate solubilization), hydrolytic enzyme production (cellulase, pectinase), biocontrol	[43]
**Municipal organic wastes**	composting	*Penicillium vinaceum* and *Eupenicillium hirayama*	*Capsicum annuum* (pepper), *Solanum melongena* (aubergine) and *S. lycopersicum* (tomato)	soil supplementation	plot tests	plant growth promotion, biocontrol	[44]
**Mixture of sawdust, sewage sludge, chicken litter**	composting	Actinomycetes (thermo-tolerant)	Okra (*Albenus Esculentus*) and Maize (*Zea-mays*)	soil amendment	plot tests	fermentation, nitrogen fixation	[34]
**composite of sawdust, chicken litter, vegetable waste, sewage sludge**	composting	*Streptomyces* spp and *Rothia* spp	Okra (*Albenus Esculentus*) and Maize (*Zea-mays*)	soil amendment	plot tests	cellulolytic, ligninolytic, nitrogen mineralization	[45]
**jarosite waste**	nanoparticle synthesis	*Aspergillus terreus* strain J4	*Triticum aestivum*	seed priming	germination tests	bioleaching	[46]
**Dairy Wastewater**	biofilm bioreactor	*Aeromonas, Bacillus, Thaueraamino aromatica, Acinetobacter* (consortium of 7 isolates)	Mung bean (var. Meha)	soil irrigation	plot tests	biofilm production	[24]
**Chicken feathers**	submerged fermentation	*Bacillus cereus*	tomato	soil amendment	plot tests	keratinolysis	[47]
**1% (w/w) keratin wastes (chicken feathers and sheep wool)**	preparation of Protein Hydrolysates (PHs)	*Trichoderma asperellum*	*Solanum lycopersicum*	weekly application of PHS	germination and pot tests	plant growth promotion (IAA production, siderophore, cellulase activity, phosphate solubilization), biocontrol	[48]
**fruit pulp from of ripened fruits not suitable for human consumption**	submerged fermentation (culture medium)	*Komagataeibacter medellinensis*	Onion	soil amendment	pot tests	production of Bacterial Nanocellulose Mulch	[49]
**Mixture of Cattle manure organic fertilizer (composted) and biochar**	fermentation	*Arthrobacter* sp. DNS10	soybean	soil amendment	germination and pot tests		[50]
**Chilli waste (stems and leaves after harvesting the fruit)**	microbial enrichment	cyanobacteria (*Fischerella muscicola, Anabaena variabilis, Aulosira fertilissima, Tolypothrix tenuis*)	Brinjal (*Solanum melongena*)	soil amendment	pot tests	nutrient recovery	[37]
**municipal wastewater (MWW) with simulated flue gas**	micro-algal biorefinery approach	*Chlorella* sp., *Dictyosphaerium* sp., *Monoraphidium* sp., *Neochloris* sp. and *Scenedesmus* sp.	Wheat	soil supplementation	Germination test	nutrient recovery	[38]
**Mixed medicinal plant waste**	composting	consortia of *Streptomyces, Paenibacillus, Bacillus* and *Hymenobacter*	*Fagopirum esculentum*, *Thymus vulgaris*, *Cynara scolimus* and *Lavandula officinalis*	seed priming	germination tests	plant growth promotion	[51]
**combination of rice washing water waste, tofu liquid waste, coconut water waste, and palm oil liquid waste**	Incubation of liquid waste with bacterial consortium for 21 days (fermentation)	*Bacillus cereus* JP6, *Bacillus cereus* JP7, *Proteus mirabilis* TKKS3, *Proteus mirabilis* TKKS7, *Providencia vermicola* SA1 and *Bacillus cereus* SA6	upland rice (*Oryza sativa* L.)	soil amendment	plot tests	cellulolysis, fermentation	[35]
**Spent coffee grounds (SCG), poultry manure, and agricultural waste-derived biochar**	composting	*Streptomyces albus, Gibellulopsis nigrescens, Bacillus licheniformis, Bacillus smithii*, and *Alternaria tenuissima*.	pepper and leek	soil amendment	germination and pot tests	microbial bioaugmentation	[52]
**sewage sludge and agricultural waste**	composting	*Bacillus megaterium*	Chinese flowering cabbage	polluted soil supplementation	plot tests	PAEs degradation and phosphate solubilization	[53]
**brewery wastewater (BWW)**	micro-algal biorefinery approach	*Scenedesmus obliquus* (ACOI 204/07)	barley and wheat seeds	soil supplementation	germination and pot tests	nutrient recovery	[54]
**cane bagasse and broadbean seed capsule composite**	solid-state fermentation (SSF)	*Kosakonia cowanii* LT-1	*Zea mays L.*	soil amendment	pot tests	EPS production	[55]
**feather waste (FW) and coconut oil cake (COC)**	Biorefinery-Fermentation	*Haloferax lucentensis* GUBF-2 MG076878	*Oryza sativa* L. var. Korgut	seed priming, soil amendment	germination and pot tests	production of protease and lipase haloextremozymes, Feather and COC Hydrolysate	[56]
**biological silage (BE) of shrimp head, molasses and milk**	fermentation	lactic acid bacteria	maralfalfa grass	application of leachate	plot tests	production of fermented liquid fertilizer	[36]
**sugarcane molasses**	fermentation	*Corynebacterium glutamicum*	*Brassica campestris* var. *Pekinensis*	foliar applications of fermented broth	pot tests	fermentation	[57]
**wastewater from a WWTP**	solid-state formulations	*Scenedesmus* sp.	Ryegrass and (b) barley	soil application of lyophilised microalgae with vegetable compost	plot tests	nutrient recovery	[58]
**pressmud**	solid state fermentation	*Bacillus circulans*	jowar and bajra	soil amendment	plot tests	fermentation and phosphate solubilization	[59]
**native chicken feathers**	fermentation	*Chryseobacterium* sp. RBT	*Solanum melongena* L and *Capsicum annuum* L.	foliar spray (S 5%, v/v) and root drenching (RD) (20%, v/v)	pot and plot tests	Keratinase	[60]
**sardine waste (SW), potato peels (PP), and poultry waste (PW)**	mesophilic bio-digestion	*Aspergillus niger* and *Saccharomyces cerevisiae*	bell peppers	soil amendment	seed germination and pot tests	fermentation	[61]
**Vermicompost and lignite (carrier)**	fermentation	*Azospirillum lipoferum* (Az 204), *Bacillus* *megaterium* (PB2) and *Pseudomonas* *fluorescens*(Pf1)	upland rice NLR 145 (*Oryza sativa* L.)	soil supplementation	pot tests	cellulolysis	[62]
**coconut fibre**	medium for mass micropropagule production	*Trichoderma asperellum* B1092	Cherry tomato var. Sakura 318	soil amendment	pot tests	biocontrol against Fusarium oxysporum f. sp. lycopersici B713T	[63]
**A. bisporus industrial wastewater**	submerged fermentation (culture medium)	*Bacillus cereus*	*Brassica chinensis* L	Application of treated liquid fermentation broth at proper intervals	plot tests	fermentation	[64]
**(ZnO)-orange peel waste composite**	bio-activation	*Bacillus* sp. AZ6	*Zea mays* L.	soil amendment	plot tests	zinc solubilization	[65]
**Lobster processing wastes**	Submerged fermentation	*Streptomyces griseus*	*Arabidopsis thaliana*	foliar applications of extracts	germination tests	chitinolysis, biocontrol	[66]
**lignocellulosic green waste (GW) from Municipal solid waste (MSW)**	composting	*Aspergillus fumigatus* and *Geotrichum* sp.	*Capsicum annuum* L.	soil amendment	pot tests	cellulolytic and ligninolytic decomposer inducer	[67]
**WWTP ash and poultry bone wastes**	granulation and microbiological activation	*Bacillus megaterium* and *Acidithiobacillus ferrooxidans*	Wheat	soil supplementation	Phytotoxicity seed germination test	phosphate solubilization	[25]
**sewage sludge ash and dried animal (porcine) blood**	biological activation and granulation	*Bacillus megaterium*	wheat (*Triticum aestivum* ssp. vulgare MacKey)	soil amendment	pot tests	phosphate solubilization	[68]
**sewage sludge ash and animal bones**	enrichment and granulation	*Bacillus megaterium* and *Acidithiobacillus ferrooxidans*	winter wheat	soil supplementation	plot tests	phosphate solubilization	[29]
**chicken feathers**	composting	*Chrysosporium indicum* JK14	*Zea mays L.*	soil amendment	pot tests	keratinolysis	[69]
**Chicken feather waste**	submerged state fermentation	*Alternaria tenuissima*	Chickpea (*Cicer arietinum*)	soil supplementation	plot tests	keratinolysis	[70]
**kitchen waste oil**	fermentation	*Pseudomonas aeruginosa* ATC 15442	Cabbage	drip irrigation	pot tests	long chain triglycerides (LCTs) degradation	[71]
**spent mushroom substrate (SMS) compost**	semi-solid fermentation	*Pantoea agglomerans* ZB	Chili pepper seedlings	soil supplementation	plot tests	phosphate solubilization	[72]
**sugarcane filter cake and boiler ash**	composting	consortia of *Pseudomonas aeruginosa* PSBR12, *Bacillus* sp. BACBR04, *Bacillus* sp. BACBR06, *Bacillus* sp. BACBR01 and *Rhizobium* sp. RIZBR01	sugarcane	soil amendment	plot tests	phosphate solubilization	[73]
**low-moisture food waste material**	composting	*Aspergillus niger* UY2015_11	*Lactuca sativa* var. *crispa* (lettuce), and *Brassica rapa* var. *perviridis*	soil supplementation	phytotoxicity and plot tests	nitrogen release, phosphate solubilization	[74]
**raw press mud**	composting	*Aspergillus niger* (RHS/M492-NAIMCC-F-02890)	*Zea mays L.*	soil amendment	plot tests	phosphate and zinc solubilization	[75]
**chicken feathers**	composting	*Bacillus subtilis* FW12	green gram	soil amendment	pot tests	keratinolysis	[76]
**kitchen waste (79%), chita-dhan (unfilled rice grain) biochar (15%), rock phosphate (5%)**	co-composting	a consortium of 10 PGPB (1%) (*Bacillus mycoides, Proteus* sp., *Bacillus cereus*, *Bacillus subtilis*, *Bacillus pumilus*, *Paenibacillus polymyxa*, and *Paenibacillus* spp.)	*Oryza sativa* L	fertilizer amendment	plot tests	plant growth promotion (IAA production, phosphate solubilization, N2 fixation)	[77]
**Glycerin pitch**	fermentation	*Lactobacillus* spp.	cucumber	soil supplementation	pot tests	glycerin biodegradation	[78]
**domestic wastewater from wastewater sewage pump with coal-fired flue gas (2.5% CO2)**	microalgae cultivation and lipid extraction	*Scenedesmus* sp.	*Oryza sativa*	soil amendment	pot tests	nutrient recovery	[79]
**organic vegetable heaps**	composting	*Clonostachys rosea f. catenula*	*Solanum lycopersicum*	soil amendment	pot tests	biocontrol, plant growth promotion	[80]
**natural phosphate and potash rock**	phosphate solubilization piles	*Acidithiobacillus thiooxidans, Beijerinckia indica*	Sugarcane	soil supplementation	plot tests	phosphate solubilization	[81]
**Crustacean shell waste (shrimp and crab shell powder (SCSP))**	Submerged fermentation	*Alcaligenes faecalis* SK10	*Pisum sativum* and *Cicer arietinum*	soil amendment	pot tests	chitinolysis and proteolysis	[82]
**vermicompost**	microbial enrichment	consortia of *Serratia marcescens, Pseudomonas aeruginosa*, and *Bacillus cereus*	tomato and wheat	soil amendment	pot tests	phosphate solubilization	[83]
**vineyard waste**	composting	*Trichoderma harzianum* T-78	muskmelon	soil supplementation	pot tests	plant growth promotion, biocontrol	[84]
**organic biomedical waste**	anerobic fermentation followed by incineration	*Pasterulla canis circinelloides*	*Solanum lycopersicum*	soil amendment	pot tests	plant nutrient dynamics, biodegradation	[85]
**mixture of feather-downy waste and dung (8 : 2)**	biohumus production through decomposition	consortium of decomposer microorganisms: *Bacillus pumilus* AL16, *Microbacterium terregens* AC1180, *Aeromonas* sp. B5376, *Arthrobacter globiformis* AC1529, *Streptomyces olivocinereus* AC1169 and *Acinetobacter* sp. B390	winter wheat crops	soil amendment	plot tests	decomposition	[28]
**sewage sludge ashes**	fungal spore inoculation for 8 days	*Penicillium bilaiae* DBS-5 and *Aspergillus niger* ATCC 9142	spring wheat (*Triticum aestivum* L. cv. Dacke)	soil application	plot tests	phosphate solubilization	[86]
**sugarcane molasses**	fermentation	*Corynebacterium glutamicum*	Potato	foliar application	plot tests	glutamic acid production	[87]
**sewage sludge ash and spent mushroom substrate**	solid-state solubilization and composting	*Acidithiobacillus ferrooxidans*	sunflower seeds	soil amendment	germination tests	phosphate solubilization	[88]
**Spent Coffee Grounds, Rice Bran, and Biochar**	composting	*Bacillus* sp. and *Actinomyces* sp.	pepper and leek	soil amendment	pot tests	plant growth, biocontrol	[89]
**biogas residue, rice straw and cattle manure**	composting	*Streptomyces microflavus* G33	*Solanum lycopersicum*	soil amendment	plot tests	biocontrol	[90]
**domestic wastewater**	microalgae cultivation and lipid extraction	*Chlorella* sp. (KP972095) and *Scenedesmus* sp. (KR025877)	*Solanum lycopersicum*	soil amendment	pot tests	nutrient recovery	[91]
**spent mushroom substrate (SMS)**	fungal digestion	*Trichoderma harzianum*	*Solanum lycopersicum*	soil amendment	pot tests	mycodegradation	[92]
**rock biofertilizers mixed with sulfur**	composting	*Acidithiobacillus, Beijerinckia indica* and *Cunninghamella elegans*	banana “Williams”	soil supplementation	plot tests	phosphate solubilization	[27]
**rice straw**	solid-state fermentation (SSF)	*Aspergillus niger* (RHS/M492-NAIMCC-F-02890)	*Oryza sativa* L	seedling spray	germination and pot tests	production of fungal-based chitosan	[93]
**black water (toilet wastewater)**	micro-algal biorefinery approach	*Chlorella sorokiniana*	spring barley (*Hordeum vulgare* L.)	soil supplementation	Phytotoxicity seed germination test and plot tests	nutrient recovery	[94]
**Chicken feathers powder**	Liquid Fermentation	*Bacillus pumilus* JYL	wheat	soil supplementation	pot tests	keratinolysis	[95]
**rice husk (RH)**	composting	*Aspergillus* sp	black gram	soil amendment	pot tests	cellulolytic activity	[39]
**paddy-soaked rice mill wastewater (PSRMW)**	micro-algal biorefinery approach	*Chlorella pyrenoidosa*	Indian okra (*Abelmoschus angulosus*)	soil amendment	pot tests	nutrient recovery	[96]
**lignin waste**	fermentation	*Meyerozyma guilliermondii* and *Providencia rettgeri*	Cowpea	foliar application	Seed germination pot tests	ligninolysis	[97]
**chicken manure**	fermentation	*Bacillus subtilis*	Chinese cabbage and rape seeds	seed immersion	germination and pot tests	decomposition	[26]
**duck feathers**	composting	consortia of *Arthrobacter ureafaciens* K10 and *Streptomyces* sp. CP3	cherry tomato	seed priming	germination and pot tests	feather degradation, phosphatesolubilization, and IAA formation	[98]
**Cow Manure and rapeseed meal**	Solid State Fermentation	*Bacillus* sp. XG-1	*Citrullus lanatus* Thumb.	soil amendment	plot tests	plant growth promotion (IAA, gibberellins, phytase), biocontrol	[99]
**prickly ash seeds (PAS) and biochar from rice husks**	solid state fermentation	*Bacillus subtilis* Tpb55	rape seeds	seed immersion and soil amendment	germination and pot tests	biocontrol	[100]

## Data Availability

Not applicable.
